# Effects of different models of sucrose intake on the oxidative status of the uterus and ovary of rats

**DOI:** 10.1371/journal.pone.0251789

**Published:** 2021-05-18

**Authors:** Joanna Sadowska, Wioleta Dudzińska, Izabela Dziaduch

**Affiliations:** 1 Department of Applied Microbiology and Human Nutrition Physiology, Faculty of Food Science and Fisheries, West Pomeranian University of Technology in Szczecin, Szczecin, Poland; 2 Institute of Biology, University of Szczecin, Szczecin, Poland; Kafrelsheikh University, EGYPT

## Abstract

The aim of the study was to assess the effect of different models of sucrose intake on carbohydrate-lipid metabolism and changes in oxidant balance in the ovaries and uterus of rats. Animals were divided into three groups: I—basic feed, II—feed contains 8% of sucrose, III—alternately every second week the basic feed and modified feed contains 16% of sucrose. The diet containing 8% of sucrose was found to result in an increased activity of antioxidant enzymes in the blood, with unchanged malonylodialdehyde concentration. Variable sucrose administration pattern intensified oxidative stress in the blood and led to disturbed redox equilibrium in the rat uterus, even at a comparable long-term sucrose uptake as in the group II. This was manifested as a reduced superoxide dismutase activity (in the blood and uterus) and a higher malonylodialdehyde concentration (in the uterus). The changes observed could have been a result of metabolic disorders (higher amount of visceral fat, higher glucose concentration, higher index of homeostasis model assessment of insulin resistance, and reduced HDL-cholesterol concentration) and endocrine disorders (higher oestrogen concentrations). Changes in the antioxidant status in the rats kept on the alternating diet, may underpin the failure of fertilised egg implantation in the uterine tissue and pregnancy completion.

## Introduction

For health reasons the World Health Organization strongly recommends limiting daily intake of free sugars to less than 10% of total energy intake [1]. But despite it becoming increasingly known that sugars can have detrimental effects on health, people persist in consuming them in excess [2–4].

Causes of excessive sugar consumption differ. The study suggests addictive effects of foods rich in simple sugars such as sucrose. The sugar stimulates the release of serotonin, endogenous opioids and dopamine in the opioid and dopaminergic systems in the brain, in the ventral tegmental area and the nucleus accumbens, colloquially termed the reward system [5]. Studies on animal models demonstrate the excessive sugar consumption to produce tolerance symptoms, whereas sugar elimination results in symptoms similar to those of withdrawal, including changes in the opioid and dopaminergic sub-systems in the reward system [6]. Excessive sugar consumption in humans may also result from succumbing to advertisements of sugar-containing products and marketing practices of food manufacturers, aimed at boosting food sales [7].

Strategies of reducing simple sugar dietary contents vary, but their efficacy being low [8]. Some subjects, following sucrose elimination from the diet, resume using sugar-rich diets and alternate sugar-free and sugar-rich diets. However, frequent alternations of diet composition may be perceived by the body as a stressor [9] which changes lipid metabolism and intensifies reactions involving free radicals [10]. There are numerous reports on the increased oxidative damage to human or experimental animal plasma, liver, skeletal muscles and heart in the course of stress [11].

The course of metabolic and free-radical reactions is affected by, *inter alia*, the form sucrose is applied in (solid or liquid sucrose-rich diet) [12]. But the effect of a different models of sucrose intake on oxidative status in ovary and uterus is still unknown. When there are deviations from reactive oxygen species (ROS) normal physiological ranges, it may lead to several diseases in these tissues [13]. Endometriosis, unexplained infertility, anovulation, and the impairment of oocyte quality may be the results of oxidative stress in the reproductive system. Therefore, a delicate balance between ROS and antioxidant potential must be kept in the ovaries and uterus [14].

In females reproduction and metabolism are precisely associated and mutually regulated [15]. Therefore, it has been hypothesised that frequent changes in the composition of the diet may be a stress factor for the organism and that by disrupting carbohydrate-lipid metabolism, they can cause oxidative stress in many tissues, including the ovaries and uterus.

Our analysis focused on the changes in red blood cells, ovaries and uterus superoxide dismutase (Cu/Zn-SOD, EC 1.15.1.1), catalase (CAT, EC 1.11.1.6), glutathione peroxidase (GPx, EC 1.11.1.9) activities and malonylodialdehyde (MDA) concentration to determine the prooxidant-antioxidant balance.

The aim of the study was to assess the effect of different models of sucrose intake on the carbohydrate-lipid metabolism and antioxidant status in the ovaries and uterus of female rats. The sucrose amount in diet was set, taking into account the WHO recommendations to limit the amount of free sugars in the diet to less than 10% of energy in the diet, but the models of its consumption was different.

## Materials and methods

### Animals and study design

The study was carried out on 33 female Wistar rats, 3 months old, with the initial body weight of 205.0 ± 16.5 g. The animals were purchased from the Department of Toxicology of Poznań University of Medical Sciences. Following a week long conditioning (drinking water and basic feed) to vivarium conditions (temp. 21–22°C, relative humidity 55–60%, light-dark cycle 12/12 h), the animals were sorted into three equinumerous groups (*n* = 11 each) of equal body weight, housed in individual cages, fed ad libitum pelleted feeds. All the protocols were approved by the Local Institutional Animal Care and Use Committee in Poznań (Approval No. 18/2015) in accordance with the European Convention for the Protection of Vertebrate Animals used for Experimental and other Scientific Purposes, Council of Europe, Strasbourg 1986.

The basic feed (BF) meet the nutrient requirements AIN-93M [16] and included most of all whole wheat and maize grain. In the modified feeds (MF1 and MF2), part of wheat grain and maize grain from the basic feed was substituted by wheat flour (type 500) and sucrose (8% or 16% of component composition which was 9.3% and 18.1% of energy diet in MF1 and MF2 respectively). The percentage of the remaining components was unchanged. Such design of fodders allows to reflect the changes taking place nowadays in the composition of diets which include simple sugars and refined carbohydrates and the way the fodders were administered mirrored modern eating behavior.

The prepared feed mixes were subjected to chemical analysis to determine the content of total nitrogen, crude fat, crude fiber, dry matter and ash [17]. Content of total nitrogen was determined by Kjeldahl method on a Kjeltec 2100 system from Foss Tecator Hillerod, Denmark and converted into an amount of protein. Crude fat was determined by Soxhlet method on a Soxtec HT6 system from Foss Tecator Hillerod Denmark; crude fiber—with the gravimetric method on ANKOM 220 Fiber Analyzer, ANKOM Technology, Macedon, NY, USA; dry matter and ash by gravimetric method on SUP-4M laboratory dryer Wawa-Med, Warsaw, Poland and muffle furnace Czylok Jastrzębie Zdrój, Poland. The content of digested carbohydrates was calculated from the difference between dry mass and the sum of the other solid ingredients. The content of gross energy and metabolic energy was estimated using the commonly applied energy equivalents [18]. The content of zinc, cooper, selenium and iron was also determined in feeds by atomic absorption spectrophotometry on ICE-3300 (Thermo Scientific, Walthom, MA, USA). The detailed component and chemical compositions of fodders used in the experiment are presented in Tables 1 and 2.

**Table 1:** Component composition of diets.

Component	Basic feed (BF)	Modified feed 1 (MF1)	Modified feed 2 (MF2)
**Wheat (g/100 g)**	36.4	6.01	6.01
**Corn grain (g/100 g)**	20.0	10.0	10.0
**Wheat bran (g/100 g)**	20.0	20.0	20.0
**Dry whey (g/100 g)**	3.0	3.0	3.0
**Fodder salt**^**1**^ **(g/100 g)**	0.3	0.3	0.3
**Soya-bean grain (g/100 g)**	17.0	17.0	17.0
**Fodder chalk**^**2**^ **(g/100 g)**	1.5	1.5	1.5
**Phosphate 2-CA**^**3**^ **(g/100 g)**	0.8	0.8	0.8
**Premix LRM**^**4**^ **(g/100 g)**	1.0	1.0	1.0
**Wheat flour (g/100 g)**	0.0	32.4	24.4
**Sucrose (g/100 g)**	0.0	8.0	16.0

^1^ Mainly NaCl

^2^- Mainly CaCO_3_

^3^- CaHPO_4_

^4^- Vitamin-mineral composition used in animals feeds content per kg: IU: A 1500000, vit. D3, 100000; mg: vit. E 8000; vit. K 300, vit. B1 1200, vit. B2 1200, vit. B6 1000, vit. B12 8, Se 100, Fe 16000, Mn 4500, Zn 6000, Cu 1300, I 100, Co 200.

**Table 2:** Chemical composition of diets.

Component	Basic feed (BF)	Modified feed 1 (MF1)	Modified feed 2 (MF2)
**Total protein (%)**	17.9 ±0.09	18.4 ±0.12	16.9 ±0.10
**% of diet energy**	21.0	21.4	19.1
**Crude fat (%)**	3.2 ±0.11	3.5 ±0.15	3.6 ±0.09
**% of diet energy**	8.58	9.28	9.05
**Carbohydrates (%)**			
**total**	63.1 ±0.55	63.9 ±0.19	65.8 ±0.48
**fiber**	6.0 ±0.15	4.4 ±0.21	4.4 ±0.13
**digested**	57.1 ±0.50	59.5 ±0.61	61.5 ±0.44
**% of diet energy**	70.4	69.3	71.8
**Dry matter (%)**	90.8 ±0.20	92.4 ±0.16	92.9 ±0.11
**Total ash (%)**	6.5 ±0.12	6.6 ±0.09	6.6 ±0.18
**Metabolizable energy**			
**(kcal/g)**	3.4 ±0.03	3.4 ±0.04	3.5 ±0.04
**(kJ/g)**	14.2 ±0.21	14.3 ±0.25	14.8 ±0.25
**Copper (mg/100 g)**	1.8 ±0.09	1.5 ±0.05	1.4 ±0.08
**Iron (mg/100 g)**	20.1 ±0.71	15.6 ±0.61	15.8 ±0.68
**Manganese (mg/100 g)**	8.0 ±0.45	7.6 ±0.49	6.7 ±0.51
**Selenium (mg/100 g)**	0.04 ±0.001	0.03 ±0.001	0.03 ±0.001
**Zinc (mg/100 g)**	9.2 ±0.52	8.6 ±0.49	7.8 ±0.61

During the experiment, group I (CG—Control Group) received the basic feed, group II (SBG—Sucrose Balanced Group) received the modified feed 1 (contain 8% of sucrose), over the whole experimental period, and group III (AFG—Alternately Fed Group) received alternately every second week the basic feed and modified feed 2 (contain 16% of sucrose)—in even week—BF, in odd week—MF2. The consumption of sucrose in groups II and III during the entire experimental period was similar and amounted to approx. 10% of the energy value of the diet. Animals from all groups received tap water to drink.

The experiment lasted for eight weeks. The amounts of consumed feeds were recorded daily and the animals were weighted once a week. On the end of the experiment rats weresacrificed and samples collected after determining that the rats were in the oestrus phase. The status of the oestrus phase was determined by examining the type and abundance of cells present in the vaginal lavage, according to the methodology described by Marcondes et al. [19]. The animals were also weighed at this time. The assigned rats were fasted overnight (12 h), and anaesthetised with an intramuscular injection (10 mg/kg b.w.) of Ketanest (Pfizer Ireland Pharmaceuticals, Ireland). The animals from the AFG group received basic feed during the last week of the experiment.

### Sample collection

Blood samples was taken from the heart (cardiac puncture) and collected into vacuum tubes (Sarstedt, Germany) with K_2_EDTA as anticoagulant, after which they were immediately centrifuged (1000 × *g*, 10 min, 4°C) and plasma was separated from erythrocytes. Next the erythrocytes were washed three times with cold phosphate-buffered saline solution (0.01 M phosphate buffer, 0.14 M NaCl, pH 7.4), and then their lysis was performed by adding 4 volumes of ice-cold high-performance liquid chromatography (HPLC)-grade water. After centrifugation (10 000 × *g*, 15 min, 4°C) supernatant was collected (erythrocytes lysate) and stored at −70°C until analysis, but not longer than for one month.

After separation, blood plasma was divided into portions and immediately deep-frozen at −70°C until analysis, but not longer than for one month.

Ovaries and uterus were collected immediately after blood samples collection. The tissue were weighed to the nearest 0.001 g and then washed with phosphate-buffered saline solution (pH 7.4), and immediately frozen in liquid nitrogen and stored at −70°C until analysis.

### Biochemical analysis

Plasma glucose (BioSystems S.A., Cat. No. 11503), triglycerides (BioSystems S.A., Cat. No. 11828), total cholesterol (BioSystems S.A., Cat. No. 11805), HDL-cholesterol (BioSystems S.A., Cat. No. 11557), LDL-cholesterol (BioSystems S.A., Cat. No. 11585) concentration were determined by the enzymatic colorimetric method on the Metertech SP-8001 spectrophotometer (Metertech, Taipei, Taiwan) according to the manufacturer’s protocol.

Insulin end estradiol (Insulin: Rat ELISA kit Demeditec Diagnostics, Kiel, Germany, Cat. No. DE2048; Estradiol: Rat ELISA kit Fine Test Wuhan Fine Biotech Co., Wuhan, China, Cat. No. ER1507) were assayed using a monoclonal antibody against rat insulin/estradiol, according to the manufacturer’s instructions. It was done using an EnVision apparatus (PerkinElmer Inc., Waltham, MA, USA).

The index of homeostasis model assessment of insulin resistance (HOMA-IR) was calculated as fasting plasma glucose [mM] and fasting plasma insulin [mU/L] divided by 22.5 [20].

### Blood analysis

Activity of CAT, SOD and GPx was measured in red blood cells lysate and expressed per gram of haemoglobin, whose concentration was measured using the Drabkin’s method (the concentration of haemoglobin was measured in hemolysates). The concentration of MDA was assayed in blood plasma.

GPx (EC 1.11.1.9) enzyme activity was measured with a GPx Assay Kit (Cayman Chemical, Ann Arbor, MI, USA, Cat. No. 703102) according to the manufacturer’s protocol. Directly before measurements, erythrocytes lysate was diluted to 1:10 using a sample buffer. The method of measuring GPx activity is based on the reaction of hydroperoxide (ROOH) reduction with reduced glutathione (GSH) catalyzed by GPx. The product of this reaction is oxidized glutathione (GSSG), which is reduced to 2 GSG in the reaction catalyzed with glutathione reductase (GR). This enzyme, by reducing GSSG to 2 GSH, at the same time oxidizes NADPH to NADP^+^, which is connected with the decrease of absorbance at the wave length of 340 nm at the temperature of 25°C. The decrease of absorbance is proportional to the activity of GPx, which is expressed as unit per millilitre.

SOD (EC 1.15.1.1) enzyme activity was measured with a SOD Assay Kit (Cayman Chemical, Ann Arbor, MI, USA, Cat. No. 706002) according to the manufacturer’s protocol. Directly before measurements, erythrocytes lysate was diluted to 1:100 using a sample buffer.

Superoxide anions generated by xanthine oxidase and hypoxanthine are detected with tetrazolium salt. The amount of enzymes needed to exhibit half of dismutation of the superoxide anions (U/ml) is defined as one unit of SOD. SOD activity is standardized using the cytochrom c and XO coupled assay. Absorbance is measured at 450 nm.

CAT (EC 1.11.1.6) enzyme activity was measured with a CAT Assay Kit (Cayman Chemical, Ann Arbor, MI, USA; Cat. No. 707002) according to manufacturer’s protocol. Directly before measurements, erythrocytes lysate was diluted to 1:1000 using a sample buffer. Measuring CAT activity is based on oxidizing methanol in the presence of an optimal concentration of hydrogen peroxide (H_2_O_2_). In the reaction formaldehyde is created, which generates colored reaction product from 4-amino-3-hydrazino-5-mercapto-1,2,4-triazole (Purpald) and are measured with spectrophotometry (540 nm). The amount of enzyme that leads to the production of 1 nmol of formaldehyde per minute (nmol/min/ml) is defined as one unit of CAT activity.

The coefficient of variation (CV) intra- and inter-assay for the tests was 5.7 and 7.2%, 3.2 and 3.7%, 3.8 and 9.9%, respectively, for the GPx, SOD and CAT assay kits.

Plasma MDA concentrations were measured with MDA Assay Kit (Wuhan EIAab Sciences Co., Ltd., Wuhan, China; Cat. No. E0597Ge) according to manufacturer’s protocol. In order to use the competitive immunoenzymatic technique with inhibition enzyme, a microplate was pre-coated with a monoclonal antibody specific for MDA. There is a competitive inhibition reaction between biotin labeled MDA and unlabeled MDA (standards or samples) pre-coated with an antibody specific for MDA. After washing off the unbound conjugate and adding avidin conjugated to Horseradish Peroxidase (HRP) substrate is added. The intensity of the achieved color is inversly proportional to the concentration of MDA in the tested sample. The CV intra- and inter-assay for the tests was <10% and <12%.

### Tissue analysis

Samples of ovarian and uterine tissue were crushed in a liquid nitrogen medium. The frozen, powdered tissue was placed in a test tube containing 500 μL of phosphate-buffered saline (pH = 7.4). Before it was cooled to 4°C, and later homogenised with a blade homogeniser (Pro Scientific, PRO200 P/N 01–02200, S/N 02–1167). The homogenates were centrifuged (10,000× *g*, 20 min, 4°C) and the supernatant that was obtained was used to assay the activities of antioxidant enzymes, malondialdehyde concentrations and total protein.

Tissue GPx, SOD, CAT and MDA were analysed using an ELISA kit—Shanghai Sunred Biological Technology Co., Ltd., Shanghai, China (GPx: Cat. No. 201-11-1705; SOD: Cat. No. 201-11-0169; CAT: Cat. No. 201-11-5106; MDA: Cat. No. 201-11-0157). We described the methods above.

Bovine serum albumin as the standard (Sigma Aldrich, St. Louis, MO, USA, Cat. No. B6916) was used to measuring the concentration of protein in each supernatant. The protein concentration was determined according to Bradford method.

### Statistical analysis

In order to assess the homogeneity of variances and normality of distribution, respectively the Levene test and a modified Shapiro-Wilk test were used. All data are expressed as means ± S.E.M. One-way ANOVA and the Tukey test using Statistica 12.0^®^ program (Statsoft, Tulsa, OK, USA) were used to analyze differences between groups. If the assumptions were not met, a logarithmic transformation was applied to the data before ANOVA. Differences between groups were considered significant when *p* ≤ 0.05.

## Results

The aim of the study was to assess the effect of different models of sucrose intake on the carbohydrate-lipid metabolism and antioxidant status in the ovaries and uterus of female rats.

An analysis of the effect of the factors used on the amount of feed intake and that of selected minerals revealed that there were no differences between groups in feed intake (335 ± 8.5 g/100 g b.w./8 weeks and 328 ± 13.8 g/100 g b.w./8 weeks and 330 ± 6.9 g/100 g b.w./8 weeks), while the intake of selected minerals differed, which was related to the different content of these components in individual feeds—higher in the BF feed–Table 3. Hence, the highest intake of all the analyzed minerals was observed in control group animals (CG), while animals from the groups fed modified feed 1 (SBG) or alternately fed group (AFG) consumed them statistically significantly less. Sucrose consumption during the entire experiment was comparable in groups SBG and AFG (26.1 ± 1.10 g/100 g b.w./8 weeks and 25.8 ± 1.04 g/100 g b.w./8 weeks). Summarizing the entire period of the experiment in SBG and AFG groups the share of sucrose in total energy intake was comparable and amounted to approx. 9.5%.

**Table 3:** Effect of sucrose content diet and alternating feeding on feed, sucrose, zinc, copper, selenium, iron and manganese intake in the examined rats.

Intake	CG	SBG	AFG
**Feed intake (g/100 g b.w./8 weeks)**	335 ± 8.5	328 ± 13.8	Σ 330 ± 6.9
			169 ± 7.8 (BF)
			161 ± 6.5 (MF2)
**Sucrose intake (g/100 g b.w./8 weeks)**	0.0 ^a^	26.1 ± 1.10 ^b^	25.8 ± 1.04 ^b^
**Zinc intake (mg/100 g b.w./8 weeks)**	30.9 ± 0.77 ^b^	28.3 ± 0.86 ^a^	28.1 ± 0.67 ^a^
**Copper intake (mg/100 g b.w./8 weeks)**	6.0 ± 0.15 ^b^	4.9 ± 0.21 ^a^	5.3 ± 0.35 ^a^
**Selenium intake (mg/100 g b.w./8 weeks)**	0.12 ± 0.003 ^b^	0.11 ± 0.004 ^a^	0.11 ± 0.003 ^a^
**Iron intake (mg/100 g b.w./8 weeks)**	67.3 ± 1.68 ^c^	51.2 ± 1.96 ^a^	59.4 ± 1.87 ^b^
**Manganese intake (mg/100 g b.w./8 weeks)**	26.7 ± 0.67 ^c^	24.9 ± 0.81 ^b^	21.8 ± 0.78 ^a^

CG—Control Group, SBG—Sucrose Balanced Group, AFG—Alternately Fed Group

^a,b,c^ -Means marked with different letters in the same line are statistically different, p ≤ 0.05.

The effect of nutrition manner on the ovarian mass was found, both in absolute values and per 100 g of body weight—Table 4. A smaller weight of this organ was found in SBG and AFG animals compared to CG ones (55.8 ± 12.20 mg and 56.2 ± 8.40 mg vs. 68.1 ± 7.01 mg). The uterine weight of the examined female rats not differ statistically significantly.

**Table 4:** Effect of sucrose content diet and alternating feeding on rat ovary and uterus weights.

Organ weight	CG	SBG	AFG
**Ovary (mg)**	68.1 ± 7.01 ^b^	56.2 ± 8.4 ^a^	55.8 ± 12.2 ^a^
**Ovary (mg/100 g b.w.)**	29.6 ± 4.54 ^b^	24.2 ± 3.3 ^a^	24.0 ± 5.3 ^a^
**Uterus (mg)**	492 ± 59.8	517 ± 88.3	531 ± 89.8
**Uterus (mg/100 g b.w.)**	217 ± 36.0	224 ± 36.5	230 ± 43.1

CG—Control Group, SBG—Sucrose Balanced Group, AFG—Alternately Fed Group

^a,b^ -Means marked with different letters in the same line are statistically different, p ≤ 0.05.

**Fig 1:**
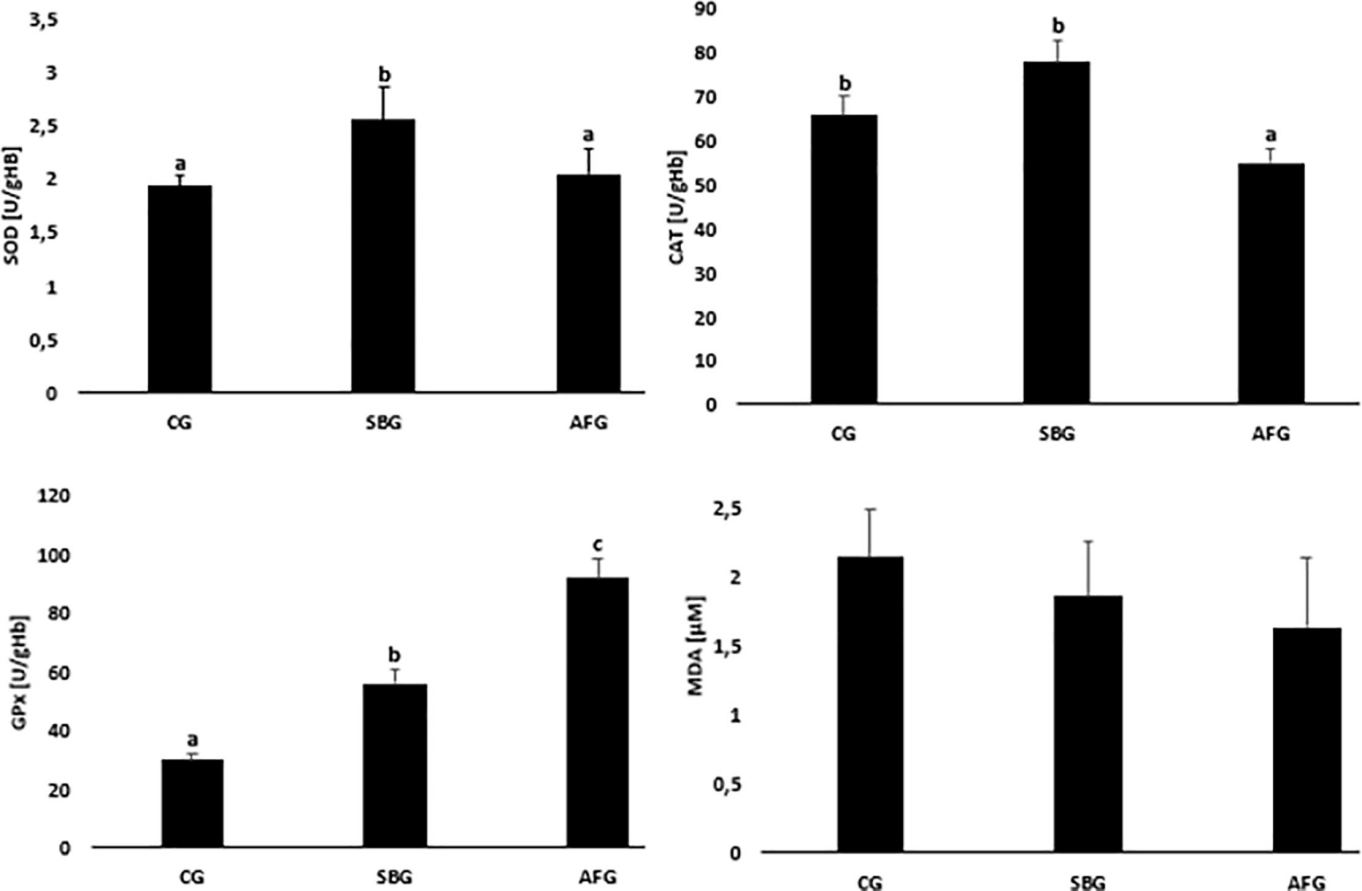
Effect of sucrose content diet and alternating feeding on erythrocyte superoxide dismutase (SOD), catalase (CAT), glutathione peroxidase (GPx) activities and plasma malonyldialdehyde (MDA) concentrations. CG—Control Group, SBG—Sucrose Balanced Group, AFG—Alternately Fed Group, ^a,b,c^ -Columns marked with different letters are statistically different, p ≤ 0.05.

Results of the activity of antioxidant defence enzymes in erythrocytes and malonyldialdehyde concentrations analyses in blood plasma of the examined animals are presented in Fig 1. In the erythrocytes of SBG animals, statistically significantly higher superoxide dismutase and glutathione peroxidase activities were found compared to CG ones (SOD 2.51 ± 0.32 U/gHb vs. 1.90 ± 0.21 U/gHb; GPx 56.2 ± 2.33 U/gHb vs. 29.8 ± 2.33 U/gHb). In animals from the AFG group the activities of superoxide dismutase and catalase were lower, while the activity of GPx was higher compared to the SBG group (SOD 2.03 ± 0.23 U/gHb vs. 2.51 ± 0.32 U/gHb; CAT 55.6 ± 3.33 U/gHb vs. 77.8 ± 5.15 U/gHb; GPx 91.8 ± 6.66 U/gHb vs. 56.2 ± 2.33 U/gHb). There were no differences in blood plasma malonyldialdehyde concentrations.

**Fig 2:**
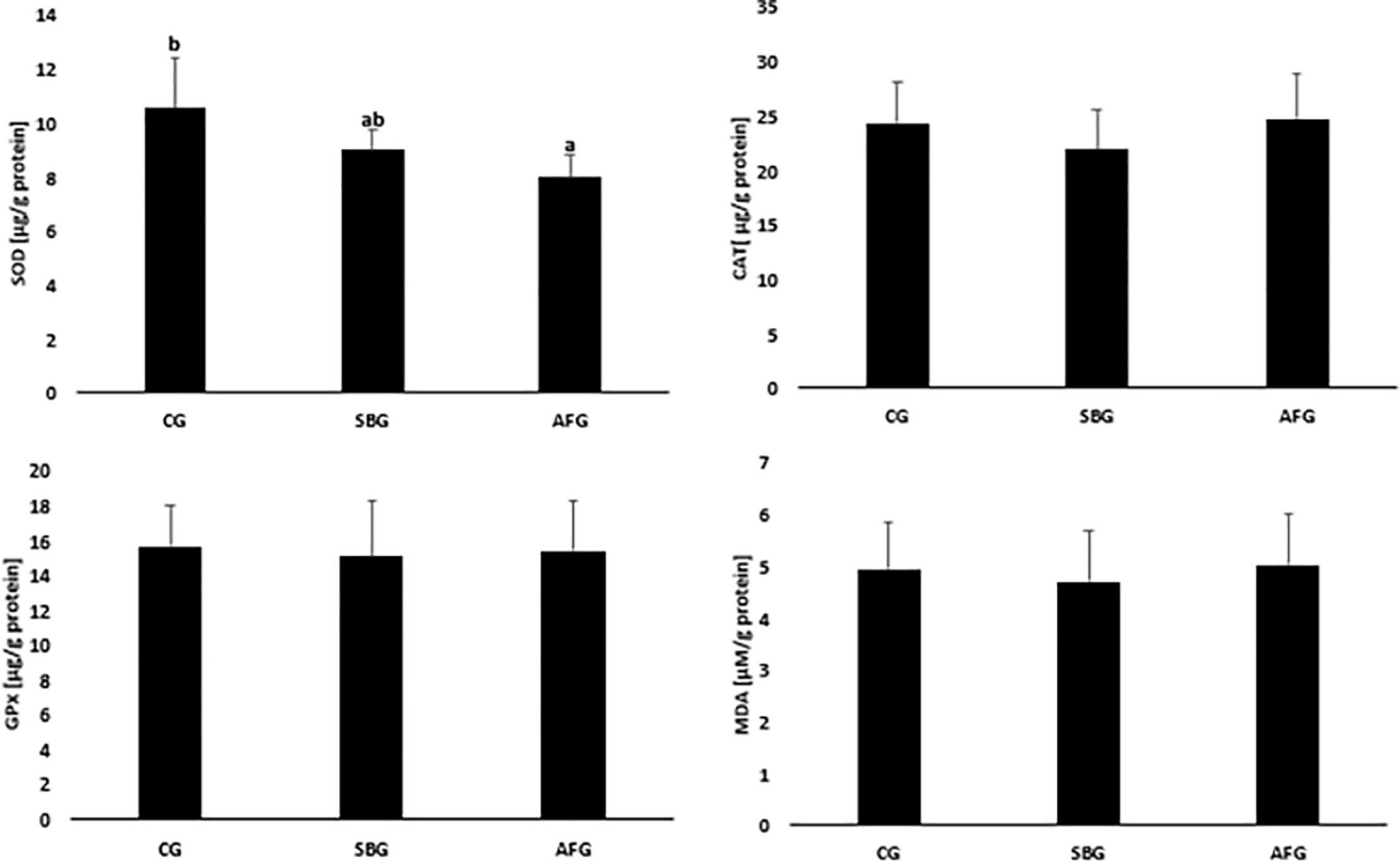
Effect of sucrose content diet and alternating feeding on ovarian superoxide dismutase (SOD), catalase (CAT), glutathione peroxidase (GPx) activities and malonyldialdehyde (MDA) concentrations. CG—Control Group, SBG—Sucrose Balanced Group, AFG—Alternately Fed Group, ^a,b^ -Columns marked with different letters are statistically different, p ≤ 0.05.

The results of analyses of the antioxidant defence enzyme activities and malonyldialdehyde concentrations in ovarian homogenates are shown in Fig 2. There were no differences in the analyzed parameters between the CG and SBG groups and the SBG and AFG groups. There was, however, a marked difference in the activity of superoxide dismutase between the CG and AFG groups. It was significantly lower in AFG animals (8.01 ± 0.81 μg/g protein vs. 10.6 ± 0.27 μg/g protein).

**Fig 3:**
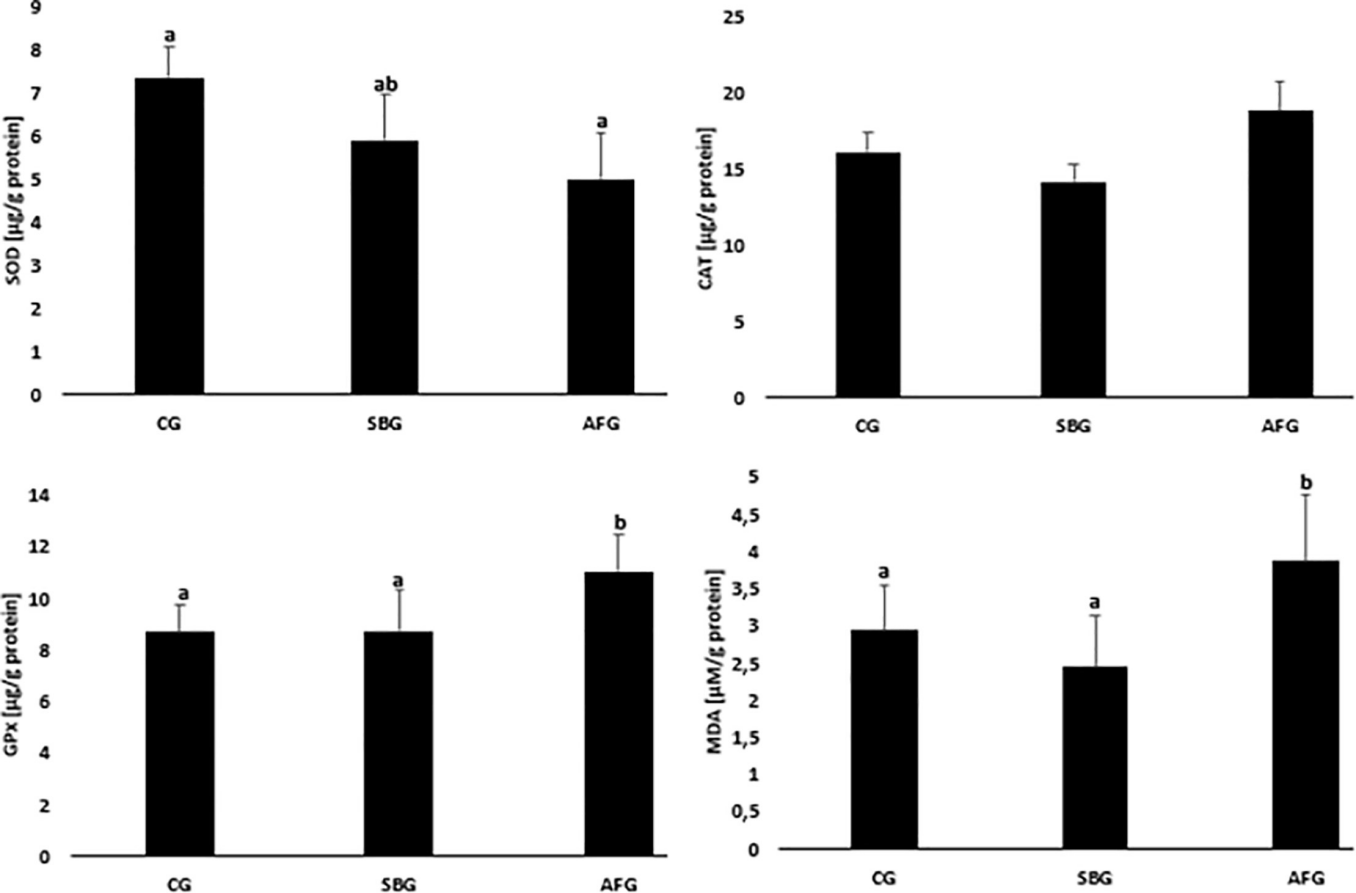
Effect of sucrose content diet and alternating feeding on uterine superoxide dismutase (SOD), catalase (CAT), glutathione peroxidase (GPx) activities and malonyldialdehyde (MDA) concentrations. CG—Control Group, SBG—Sucrose Balanced Group, AFG—Alternately Fed Group, ^a,b^ -Columns marked with different letters are statistically different, p ≤ 0.05.

The results of the analyses of the effect of the factors used on the redox balance in uterine homogenates are presented in Fig 3. When analysed the obtained results we observed that there were no differences in the analyzed parameters between the CG and SBG groups. In the uterine homogenates from AFG animals statistically significantly higher glutathione peroxidase activity and MDA concentration were found compared to SBG ones (GPx 11.05 ± 1.38 μg/g protein vs. 8.8 ± 1.56 μg/g protein; MDA 3.9 ± 0.90 μM/g protein vs. 2.4 ± 0.70 μM/g protein) and lower superoxide dismutase activity compared to CG animals (5.01 ± 1.08 μg/g protein vs. 7.4 ± 0.73 μg/g protein).

Glucose concentration was statistically significantly higher in AFG animals than in CG and SBG ones (5.60 ± 0.68 mmol/L vs. 4.56 ± 0.54 mmol/L and 4.91 ± 0.41 mmol/L)—Table 5. Insulin concentration was statistically significantly higher in SBG group compared to CG and AFG groups (3.29 ± 1.05 vs. 2.51 ± 0.89 mU/mL and 2.73 ± 0.91 mU/mL). The HOMA-IR index value was also statistically significantly different between grups, it was higher in SBG and AFG animals compared to CG ones (0.72 ± 0.18 and 0.68 ± 0.23 vs. 0.51 ± 0.11).

**Table 5:** Effect of sucrose content diet and alternating feeding on glucose, insulin, lipid concentrations and HOMA-IR value in the examined rats.

Traits	CG	SBG	AFG
**Glucose (mmol/L)**	4.56 ± 0.54 ^a^	4.91 ± 0.41 ^a^	5.60 ± 0.68 ^b^
**Insulin (mU/mL)**	2.51 ± 0.89 ^a^	3.29 ± 1.05 ^b^	2.73 ± 0.91 ^a^
**HOMA-IR**	0.51 ± 0.11 ^a^	0.72 ± 0.18 ^b^	0.68 ± 0.23 ^b^
**TG (mmol/L)**	0.48 ± 0.05 ^a^	0.45 ± 0.09 ^a^	0.61 ± 0.08 ^b^
**TC (mmol/L)**	2.03 ± 0.16 ^b^	2.08 ± 0.20 ^b^	1.71 ± 0.19 ^a^
**HDL-C (mmol/L)**	1.21 ± 0.08 ^c^	1.25 ± 0.09 ^b^	0.76 ± 0.08 ^a^
**LDL-C (mmol/L)**	0.58 ± 0.03 ^a^	0.54 ± 0.06 ^a^	0.72 ± 0.08 ^b^
**Oestrogens (pg/mL)**	14.3 ± 1.84 ^a^	22.5 ± 1.95 ^b^	21.1 ± 2.52 ^b^

TG—Triglycerides, TC—Total cholesterol, HDL-C—HDL-cholesterol, LDL-C—LDL-cholesterol, CG—Control Group, SBG—Sucrose Balanced Group, AFG—Alternately Fed Group

a,b,c—Means marked with different letters in the same line are statistically different, p ≤ 0.05.

The influence of the factors used was found on concentrations of blood lipid parameters–Table 5. The concentration of triglycerides was higher in AFG compared to CG and SBG groups (0.61 ± 0.08 mmol/L vs 0.48 ± 0.05 mmol/L and 0.45 ± 0.09 mmol/L). However, the total cholesterol level was statistically significantly lower in AFG animals compared to CG and SBG ones (1.71 ± 0.19 mmol/L vs. 2.03 ± 0.16 mmol/L and 2.08 ± 0.20 mmol/L). This was associated with a statistically significantly lower HDL-cholesterol and higher LDL-cholesterol concentration.

The applied experimental factors also influenced the concentration of estrogens—Table 5. Their higher concentrations were found in the groups fed with modified feeds compared to that fed with control feed (22.5 ± 1.95 pg/mL and 21.1 ± 2.52 pg/mL vs. 14.3 ± 1.84 pg/mL).

Weight gains stated during the experiment were statistically significantly higher in SBG and AFG animals compared to CG one (27.7 ± 2.51 g and 26,8 ± 3.13 g vs. 22.7 ± 2.74 g)—Table 6. But in SBG they were not associated with the deposition of visceral fat, because its highest amount was found only in AFG animals, which accumulated it statistically significantly more compared to CG and SBG animals, both in absolute values and per 100 g body weight (3.77 ± 0.35 g b.w. vs. 2.99 ± 0.37 g b.w. and 3.02 ± 0.29 g/100 g b.w.).

**Table 6:** Effect of sucrose content diet and alternating feeding on body weight gain and visceral fat in the examined rats.

Traits	CG	SBG	AFG
**Body weight gain (g)**	22.75 ± 2.74 ^a^	27.7 ± 2.51 ^b^	26.8 ± 3.13 ^b^
**Visceral fat (g)**	6.62 ± 0.48 ^a^	7.04 ± 0.52 ^a^	8.75 ± 0.68 ^b^
**Visceral fat (g/100 g b.w.)**	2.99 ± 0.37 ^a^	3.02 ± 0.29 ^a^	3.77 ± 0.35 ^b^

CG—Control Group, SBG—Sucrose Balanced Group, AFG—Alternately Fed Group

a,b—Means marked with different letters in the same line are statistically different, p ≤ 0.05.

## Discussion

Results of the study point to a relationship between the presence of sucrose in the diet and a model of its application and the oxidant-antioxidant equilibrium in the female rat blood and reproductive organs.

Erythrocytes of the individuals consistently supplied with sucrose at a level of 8% of diet (group SBG) showed a significantly higher activity of SOD and GPx, despite a significantly lower consumption of minerals involved in synthesis of the enzymes. A higher activity of antioxidant enzymes may be indicative of strengthened defence mechanisms which protect cells from negative effects of ROX, *inter alia* lipid peroxidation, as indicated by the absence of changes in the MDA concentration.

At the nutrition model involving alternating periods of sucrose-free and sucrose-rich (group AFG) diets, the activity of SOD and CAT was lower, the GPx activity remaining increased, which may result in the absence of effective antioxidant defence in cells and tissues. This may be indicative of intensified ROS production and antioxidant defence being strengthened until the potential for SOD and CAT synthesis becomes exhausted. The blood antioxidant potential was maintained, as indicated by the lack of changes in the MDA concentration, most probably due to a higher GPx activity.

Also Maciejczyk [21] and Sadowska et al. [22] reported an increased ROS production and intensified blood and hepatic antioxidant processes in rats fed sucrose-containing diets. In addition, Jarukamjorn et al. [23] demonstrated temporal changes in the blood and tissue antioxidant status in animals fed sucrose-containing diets. In their study, the high-sucrose diet resulted initially in an intensified antioxidant defence manifested as increased SOD, CAT and GPx activities. However, in week 8 of the experiment, activities of SOD and CAT were observed to decline, the GPx activity undergoing small changes only. However, Busserolles et al. [24] showed the sucrose-rich diet to result, as early as after 2 weeks, in an increased lipid peroxidation product concentration (as measured with the TBARS concentration) and a reduced SOD activity, the GPx activity remaining unchanged. Busserolles et al. [24] demonstrated that even a short-term application of a sucrose-rich diet adversely affects the redox equilibrium, which suggests that metabolic disorders accompanying a sucrose-rich diet may be associated with oxidative stress, particularly if consumption of dietary antioxidants is low. Data reported by various authors are, however, difficult to compare on account of different dietary sucrose levels used. The sucrose contribution to dietary energy in high-sucrose diets ranged within 15–40%, whereas in the MF1 diet applied in this study the sucrose content was 8%; hence, this diet cannot be regarded as a high-sucrose one. It may be seen, however, that even such low amounts of dietary sucrose disturb the blood oxidant-antioxidant equilibrium.

Changes in antioxidant enzyme activity in the blood of the AFG individuals, observed in this study and indicative of a disturbed oxidant-antioxidant equilibrium, could have been produced by a higher blood plasma glucose concentration. Hyperglycaemia initiates numerous changes influencing cellular metabolic disorders, including the redox equilibrium, *inter alia* via intensification of oxidation processes in mitochondria, an intensified NADPH oxidase [EC 1.6.3.1] activity; intensified non-enzymatic glycation of proteins, lipids, and nucleic acids; protein kinase C activation; formation of glycosaminoglycans; and activation of the nuclear transcription factor (NFKB) responsible for, *inter alia*, development of inflammatory reactions [25, 26]. Oxidative stress could have been additionally strengthened by a higher accumulation of visceral adipose tissue found in that group, compared to groups CG and SBG. The visceral adipose tissue, constituting a source of pro-inflammatory cytokines [27], is capable of generating ROS via inflammatory processes [28]. Important could have also been the HDL-cholesterol level, lower than that in other groups. High-density proteins have the antioxidant properties resulting of the presence of apo A-I protein and enzymes such as 1-sphingosine phosphate, platelet-activating acetyl hydrolase and paraoxonase I, and are capable of immobilising lipid peroxidation products [29, 30]. The SBG and AFG individuals showed also a reduced insulin sensitivity, manifested as a significant increase in HOMA-IR, the values of which are positively correlated with increased ROS production and antioxidant defence intensification [31]. An excessive ROS synthesis and deficient antioxidant defence act in favour of insulin resistance [32] and, consequently, poor availability of glucose in cells. The poor glucose availability, or the absence of glucose altogether, may alter preferential oxidation of the available energy substrates and intensify metabolism of fatty acids as an alternative energy substrate [33]. The result is an intensified formation of NADH and FADH2 dinucleotides, major energy vectors for ATP production. During the intensive electron transport, some electrons leave the main reaction pathway to initiate superoxide anion (O2^-^) generation. Oxidative stress within the AFG individuals could have been thus a consequence of an excessive ROS generation, a reduced efficiency of natural antioxidant systems, or both.

Results of our studies showed that alternation of sucrose-free and sucrose-rich diets intensified oxidative stress and disturbed the redox homeostasis, compared to the diet in which sucrose contributed 9.3% to the energy content, even at a similar long-term sucrose uptake (26.1 vs. 25.8 g/100 b.w./8 weeks). The AFG animals showed reduced activities of SOD and CAT at a higher activity of GPx, the uterus showing a higher concentration of MDA (a cellular oxidative stress indicator), which points to an intensified ROS production in the face of unsatisfactory antioxidant protection.

The emergent oxidative stress may result in structural changes in proteins, lipids, carbohydrates, and nucleic acids. ROS bring about oxidative modification of LDL lipoproteins, intensify oxidative modification of LDL lipoproteins, cause damage and remodeling of blood vessels and increase expression of ICAM-1 and VCAM-1 adhesive molecules [34]. This increases the risk of cardiac-vascular diseases such as atherosclerosis and aortal hypertension. In our study, changes in antioxidant enzymes in the blood and tissues of the AFG rats were accompanied by adverse changes in blood lipid parameters: higher concentrations of TG and LDL-C as well as a decreased concentration of HDL-C. Such a profile of blood lipid parameter changes is particularly disadvantageous because low HDL concentrations are a significant atherosclerosis risk factor [30, 35], additionally enhanced by oxidative stress in the animals studied.

Our study showed oxidative stress in the AFG rats to be visible not only in the blood, but also in the uterus and ovaries, which may, in consequence, result in female fecundity disorders. Uterine homogenates from animals fed alternately sucrose-free and sucrose-rich diets showed a lower SOD activity, compared to the CG animals, as well as a higher GPx activity and MDA concentration, compared to the SBG rats. Disturbances in the redox equilibrium in the uterus were thus more intense in the AFG animals, compared to the SBG ones. The results show the uterus and ovary to differ considerably in the oxidative defence status and the amount of ROS generated. The AFG rat uterus showed changed activities of the antioxidant defence enzymes and intensified lipid peroxidation processes, confirmed by the higher MDA concentration. On the other hand, the CAT and GPx activities as well as the MDA concentration in ovaries of that group were comparable to those determined in the remaining groups, whereas the SOD activity was lower, but only with respect to the control.

The difference in the amount of ROS generated and in the performance of antioxidant mechanism between the uterus and ovaries were confirmed also by Sadowska et al. [22] and Farombi et al. [36]. Ovaries seem to be primarily protected from free radicals. Farombi et al. [36] showed that the H_2_O_2_ concentration, which is a marker of intensified free radical reaction, remain unchanged in the ovaries, but was significantly greater in the uterus.

In the analysed tissues, the redox status could have been affected by oestrogens. They play an important part in maintaining the tissue redox balance by controlling the mitochondrial ROS level [37]. But their anti free-radical activity is marked in tissues in which dominate beta oestrogen receptors, characteristic for the ovaries, but not for the uterus, in which dominate alpha oestrogen receptors [38–40]. This may explain the absence of oxidative stress in the SBG and AFG ovaries.

Higher blood oestrogen concentrations in the SBG and SFG rats could have resulted from a higher body weight increase during the experiment and, as a result, a higher final body weight, compared to the control. Body weight has been shown to be directly correlated with the oestrogen concentration [41]. The excessive body weight and the correlated increase in the oestrogen concentration could result in cessation of ovulation [42]. This is a result of the FSH release being inhibited at an excessive oestrogen content [43]. Obesity brings about disturbances in fecundity not only via an increased oestrogen concentration, but also as a consequence of higher insulin concentration and insulin resistance, which was exposed as an increased HOMA-IR [44].

To maintain the regular tissue function, necessary is the equilibrium between the amount of emergent ROS and the antioxidant defence. An adequate ROS level and its scavenging systems play an important role in various processes of reproductive physiology and fertility [45, 46] including follicular development [14], oocyte maturation [47] and ovulation [48]. They make it also possible to maintain endocrine equilibrium via, *inter alia*, inhibition of progesterone synthesis at the end of the luteal phase of the cycle [49]. On the other hand, oxidative stress in the uterus may be one of the causes of repeated embryo implantation failure and recurrent abortions [36].

### Limitations

In order to verify the current findings and more fully examine the mechanisms underlying our observations, it is necessary to extend the research to identify and explain the relationship between the observed changes and 1) the level of pro-inflammatory cytokines, especially in the AFG group, where an increase in visceral adipose tissue was observed; 2) the level of oestrogen receptors and the ratio of ERα to Erβ in the ovaries and uterus; 3) increase in oestrogen concentration and decrease in ovarian weight in the SBG and AFG groups and the reasons for the lower weight of the ovaries in the test groups should be clarified.

## Conclusions

To sum up, the diet containing a 8% of sucrose (9.3% contribution of sucrose to the energy content) was found to result in an intensification of the free-radical-based process, compensated by an increased activity of antioxidant enzymes in the blood. Alternation, at a week-long interval, of sucrose-free and sucrose-rich diets intensified oxidative stress in the blood and led to disturbed redox equilibrium in the rat uterus, even at a comparable long-term sucrose uptake, such as that in the SBG rats. The changes observed could have been a result of metabolic disorders (a higher amount of visceral fat tissue, a higher glucose concentration, a higher HOMA-IR, and a reduced HDL-C concentration) as well as endocrine disorders (higher oestrogen concentrations).

## Supporting information

S1 Table Effect of sucrose content diet and alternating feeding on feed, sucrose, zinc, copper, selenium, iron and manganese intake in the examined rats. CG—Control Group, SBG—Sucrose Balanced Group, AFG—Alternately Fed Group.
(DOCX)

S2 Table Effect of sucrose content diet and alternating. CG—Control Group, SBG—Sucrose Balanced Group, AFG—Alternately Fed Group.
(DOCX)

S3 Table Effect of sucrose content diet and alternating feeding on glucose, insulin, lipid concentrations and HOMA-IR value in the examined rats. CG—Control Group, SBG—Sucrose Balanced Group, AFG—Alternately Fed Group.
(DOCX)

S4 Table Effect of sucrose content diet and alternating feeding on body weight gain and visceral fat in the examined rats. CG—Control Group, SBG—Sucrose Balanced Group, AFG—Alternately Fed Group.
(DOCX)

S5 Table Effect of sucrose content diet and alternating feeding on erythrocyte superoxide dismutase (SOD), catalase (CAT), glutathione peroxidase (GPx) activities and plasma malonyldialdehyde (MDA) concentrations. CG—Control Group, SBG—Sucrose Balanced Group, AFG—Alternately Fed Group.
(DOCX)

S6 Table Effect of sucrose content diet and alternating feeding on ovarian superoxide dismutase (SOD), catalase (CAT), glutathione peroxidase (GPx) activities and malonyldialdehyde (MDA) concentrations. CG—Control Group, SBG—Sucrose Balanced Group, AFG—Alternately Fed Group.
(DOCX)

S7 Table Effect of sucrose content diet and alternating feeding on uterine superoxide dismutase (SOD), catalase (CAT), glutathione peroxidase (GPx) activities and malonyldialdehyde (MDA) concentrations. CG—Control Group, SBG—Sucrose Balanced Group, AFG—Alternately Fed Group.
(DOCX)
