# Supplementary material for: Effects of different models of sucrose intake on the oxidative status of the uterus and ovary of rats
Source: PLoS One. 2021 May 18;16(5):e0251789. doi: 10.1371/journal.pone.0251789 (PMC8130931; doi:10.1371/journal.pone.0251789)
Supplement: S1 Table — CG—Control Group, SBG—Sucrose Balanced Group, AFG—Alternately Fed Group. (DOCX) [file pone.0251789.s001.docx]

| **S1 Table.**  Effect of sucrose content diet and alternating feeding on feed, sucrose, zinc, copper, selenium, iron and manganese intake in the examined rats. | | | | |
| --- | --- | --- | --- | --- |
|  |  | **CG (n=11)** | **SBG (n=11)** | **AFG (n=11)** |
| **Feed intake**  **(g/100 g b.w./8 weeks)** | **Mean** | 335 | 328 | 330 |
|  | **SD** | ±8.5 | ±13.8 | ±6.9 |
|  | **Min.** | 319 | 306 | 318 |
|  | **Max.** | 349 | 349 | 338 |
|  | **Median** | 335 | 335 | 330 |
| **Sucrose intake**  **(g/100 g b.w./8 weeks)** | **Mean** | 0.0 | 26.1 | 28.1 |
|  | **SD** | ±0.0 | ±1.10 | ±0.67 |
|  | **Min.** | 0.0 | 24.2 | 23.7 |
|  | **Max.** | 0.0 | 27.4 | 26.9 |
|  | **Median** | 0.0 | 26.4 | 26.1 |
| **Zinc intake**  **(mg/100 g b.w./8 weeks)** | **Mean** | 30.9 | 28.3 | 28.1 |
|  | **SD** | ±0.77 | ±0.86 | ±0.67 |
|  | **Min.** | 29.3 | 27.2 | 26.8 |
|  | **Max.** | 32.1 | 29.3 | 28.8 |
|  | **Median** | 30.8 | 28.8 | 28.1 |
| **Copper intake**  **(mg/100 g b.w./8 weeks)** | **Mean** | 6.0 | 4.9 | 5.3 |
|  | **SD** | ±0.15 | ±0.21 | ±0.35 |
|  | **Min.** | 5.7 | 4.5 | 4.5 |
|  | **Max.** | 6.3 | 5.2 | 6.1 |
|  | **Median** | 6.0 | 5.0 | 5.3 |
| **Selenium intake**  **(mg/100 g b.w./8 weeks)** | **Mean** | 0.12 | 0.11 | 0.11 |
|  | **SD** | ±0.003 | ±0.004 | ±0.003 |
|  | **Min.** | 0.120 | 0.110 | 0.106 |
|  | **Max.** | 0.128 | 0.122 | 0.119 |
|  | **Median** | 0.125 | 0.117 | 0.115 |
| **Iron intake**  **(mg/100 g b.w./8 weeks)** | **Mean** | 67.3 | 51.2 | 59.4 |
|  | **SD** | ±1.68 | ±1.96 | ±1.87 |
|  | **Min.** | 63.7 | 48.1 | 54.9 |
|  | **Max.** | 69.6 | 53.6 | 61.1 |
|  | **Median** | 67.3 | 52.2 | 59.5 |
| **Manganese intake**  **(mg/100 g b.w./8 weeks)** | **Mean** | 26.7 | 24.9 | 21.8 |
|  | **SD** | ±0.67 | ±0.81 | ±0.78 |
|  | **Min.** | 25.2 | 24.3 | 20.5 |
|  | **Max.** | 27.5 | 27.3 | 22.9 |
|  | **Median** | 26.8 | 24.8 | 21.8 |

CG - Control Group, SBG - Sucrose Balanced Group, AFG - Alternately Fed Group,
