# Supplementary material for: Effects of different models of sucrose intake on the oxidative status of the uterus and ovary of rats
Source: PLoS One. 2021 May 18;16(5):e0251789. doi: 10.1371/journal.pone.0251789 (PMC8130931; doi:10.1371/journal.pone.0251789)
Supplement: S2 Table — CG—Control Group, SBG—Sucrose Balanced Group, AFG—Alternately Fed Group. (DOCX) [file pone.0251789.s002.docx]

| **S2 Table.**  Effect of sucrose content diet and alternating feeding on rat ovary and uterus weights. | | | | |
| --- | --- | --- | --- | --- |
|  |  | **CG (n=11)** | **SBG (n=11)** | **AFG (n=11)** |
| **Ovary (mg)** | **Mean** | 68.1 | 56.2 | 55.8 |
|  | **SD** | ±7.01 | ±8.4 | ±12.2 |
|  | **Min.** | 57.0 | 45.0 | 39.0 |
|  | **Max.** | 76.0 | 71.0 | 74.0 |
|  | **Median** | 71.0 | 55.0 | 53.0 |
| **Ovary**  **(mg/100 g b.w.)** | **Mean** | 29.6 | 24.2 | 24.0 |
|  | **SD** | ±4.54 | ±3.3 | ±5.3 |
|  | **Min.** | 19.9 | 19.5 | 17.3 |
|  | **Max.** | 35.7 | 29.9 | 33.2 |
|  | **Median** | 29.3 | 24.5 | 23.4 |
| **Uterus (mg)** | **Mean** | 492 | 517 | 531 |
|  | **SD** | ±59.7 | ±88.3 | ±89.8 |
|  | **Min.** | 410 | 385 | 415 |
|  | **Max.** | 619 | 643 | 704 |
|  | **Median** | 482 | 488 | 499 |
| **Uterus**  **(mg/100 g b.w.)** | **Mean** | 217 | 224 | 230 |
|  | **SD** | ±36.0 | ±36.5 | ±43.1 |
|  | **Min.** | 165 | 175 | 174 |
|  | **Max.** | 294 | 281 | 328 |
|  | **Median** | 208 | 210 | 218 |

CG - Control Group, SBG - Sucrose Balanced Group, AFG - Alternately Fed Group,
