# Supplementary material for: Effects of different models of sucrose intake on the oxidative status of the uterus and ovary of rats
Source: PLoS One. 2021 May 18;16(5):e0251789. doi: 10.1371/journal.pone.0251789 (PMC8130931; doi:10.1371/journal.pone.0251789)
Supplement: S3 Table — CG—Control Group, SBG—Sucrose Balanced Group, AFG—Alternately Fed Group. (DOCX) [file pone.0251789.s003.docx]

| **S3 Table.**  Effect of sucrose content diet and alternating feeding on glucose, insulin, lipid concentrations and HOMA-IR value in the examined rats. | | | | |
| --- | --- | --- | --- | --- |
|  |  | **CG (n=11)** | **SBG (n=11)** | **AFG (n=11)** |
| **Glucose**  **(mmol/L)** | **Mean** | 4.56 | 4.91 | 5.60 |
|  | **SD** | ±0.54 | ±1.05 | ±0.91 |
|  | **Min.** | 3.75 | 4.21 | 4.96 |
|  | **Max.** | 5.88 | 5.60 | 6.65 |
|  | **Median** | 4.61 | 4.83 | 5.45 |
| **Insulin**  **(mU/mL)** | **Mean** | 2.51 | 3.29 | 2.73 |
|  | **SD** | ±0.89 | ±1.05 | ±0.91 |
|  | **Min.** | 1.10 | 2.11 | 1.35 |
|  | **Max.** | 3.96 | 5.30 | 3.96 |
|  | **Median** | 2.35 | 3.25 | 2.60 |
| **HOMA-IR** | **Mean** | 0.51 | 0.72 | 0.68 |
|  | **SD** | ±0.11 | ±0.18 | ±0.23 |
|  | **Min.** | 0.31 | 0.44 | 0.32 |
|  | **Max.** | 0.65 | 1.04 | 1.16 |
|  | **Median** | 0.51 | 0.75 | 0.67 |
| **TG**  **(mmol/L)** | **Mean** | 0.48 | 0.45 | 0.61 |
|  | **SD** | ±0.05 | ±0.09 | ±0.08 |
|  | **Min.** | 0.40 | 0.34 | 0.45 |
|  | **Max.** | 0.58 | 0.57 | 0.70 |
|  | **Median** | 0.48 | 0.48 | 0.63 |
| **TC**  **(mmol/L)** | **Mean** | 2.03 | 2.08 | 1.71 |
|  | **SD** | ±0.16 | ±0.20 | ±0.19 |
|  | **Min.** | 1.80 | 1.73 | 1.46 |
|  | **Max.** | 2.26 | 2.43 | 2.09 |
|  | **Median** | 2.06 | 2.08 | 1.71 |
| **HDL-C**  **(mmol/L)** | **Mean** | 1.21 | 1.25 | 0.76 |
|  | **SD** | ±0.08 | ±0.09 | ±0.08 |
|  | **Min.** | 1.08 | 1.01 | 0.60 |
|  | **Max.** | 1.33 | 1.38 | 0.93 |
|  | **Median** | 1.20 | 1.25 | 0.76 |
| **LDL-C**  **(mmol/L)** | **Mean** | 0.58 | 0.54 | 0.72 |
|  | **SD** | ±0.03 | ±0.06 | ±0.08 |
|  | **Min.** | 0.52 | 0.43 | 0.56 |
|  | **Max.** | 0.61 | 0.61 | 0.91 |
|  | **Median** | 0.58 | 0.56 | 0.73 |
| **Oestrogens**  **(pg/mL)** | **Mean** | 14.3 | 22.5 | 21.1 |
|  | **SD** | ±1.84 | ±1.95 | ±2.52 |
|  | **Min.** | 11.8 | 19.5 | 17.8 |
|  | **Max.** | 17.4 | 26.2 | 25.6 |
|  | **Median** | 14.0 | 22.5 | 21.2 |

CG - Control Group, SBG - Sucrose Balanced Group, AFG - Alternately Fed Group,
