# Supplementary material for: Effects of different models of sucrose intake on the oxidative status of the uterus and ovary of rats
Source: PLoS One. 2021 May 18;16(5):e0251789. doi: 10.1371/journal.pone.0251789 (PMC8130931; doi:10.1371/journal.pone.0251789)
Supplement: S4 Table — CG—Control Group, SBG—Sucrose Balanced Group, AFG—Alternately Fed Group. (DOCX) [file pone.0251789.s004.docx]

| **S4 Table.**  Effect of sucrose content diet and alternating feeding on body weight gain and visceral fat in the examined rats. | | | | |
| --- | --- | --- | --- | --- |
|  |  | **CG (n=11)** | **SBG (n=11)** | **AFG (n=11)** |
| **Body weight gain**  **(g)** | **Mean** | 22.8 | 27.7 | 26.8 |
|  | **SD** | ±2.74 | ±2.52 | ±3.13 |
|  | **Min.** | 19.6 | 24.6 | 23.6 |
|  | **Max.** | 28.8 | 31.2 | 31.2 |
|  | **Median** | 22.2 | 26.9 | 25.4 |
| **Visceral fat (g)** | **Mean** | 6.62 | 7.04 | 8.75 |
|  | **SD** | ±0.48 | ±0.52 | ±0.68 |
|  | **Min.** | 6.01 | 6.43 | 7.88 |
|  | **Max.** | 7.80 | 7.80 | 9.82 |
|  | **Median** | 6.64 | 6.95 | 8.79 |
| **Visceral fat**  **(g/100 g b.w.)** | **Mean** | 2.99 | 3.02 | 3.77 |
|  | **SD** | ±0.37 | ±0.29 | ±0.35 |
|  | **Min.** | 2.64 | 2.60 | 3.26 |
|  | **Max.** | 3.96 | 3.65 | 4.49 |
|  | **Median** | 2.95 | 3.04 | 3.74 |

CG - Control Group, SBG - Sucrose Balanced Group, AFG - Alternately Fed Group,
