# Supplementary material for: Effects of different models of sucrose intake on the oxidative status of the uterus and ovary of rats
Source: PLoS One. 2021 May 18;16(5):e0251789. doi: 10.1371/journal.pone.0251789 (PMC8130931; doi:10.1371/journal.pone.0251789)
Supplement: S5 Table — CG—Control Group, SBG—Sucrose Balanced Group, AFG—Alternately Fed Group. (DOCX) [file pone.0251789.s005.docx]

| **S5 Table.**  Effect of sucrose content diet and alternating feeding on erythrocyte superoxide dismutase (SOD), catalase (CAT), glutathione peroxidase (GPx) activities and plasma malonyldialdehyde (MDA) concentrations. | | | | |
| --- | --- | --- | --- | --- |
|  |  | **CG (n=11)** | **SBG (n=11)** | **AFG (n=11)** |
| **SOD**  **(U/gHb)** | **Mean** | 1.90 | 2.51 | 2.03 |
|  | **SD** | ±0.21 | ±0.32 | ±0.23 |
|  | **Min.** | 1.55 | 1.86 | 1.64 |
|  | **Max.** | 2.29 | 3.07 | 2.41 |
|  | **Median** | 1.89 | 2.51 | 2.05 |
| **GPx**  **(U/gHb)** | **Mean** | 29.85 | 56.25 | 91.86 |
|  | **SD** | ±2.33 | ±2.33 | ±6.66 |
|  | **Min.** | 24.45 | 52.32 | 81.39 |
|  | **Max.** | 32.18 | 61.46 | 101.99 |
|  | **Median** | 30.67 | 55.98 | 90.61 |
| **CAT**  **(U/gHb)** | **Mean** | 65.74 | 77.81 | 55.63 |
|  | **SD** | ±4.45 | ±5.15 | ±3.33 |
|  | **Min.** | 58.64 | 66.11 | 50.28 |
|  | **Max.** | 73.80 | 84.92 | 61.29 |
|  | **Median** | 65.86 | 76.89 | 55.44 |
| **MDA**  **(µmol/L)** | **Mean** | 2.09 | 1.86 | 1.70 |
|  | **SD** | ±0.31 | ±0.40 | ±0.45 |
|  | **Min.** | 1.69 | 1.00 | 0.99 |
|  | **Max.** | 2.80 | 2.67 | 1.87 |
|  | **Median** | 2.15 | 1.87 | 1.63 |

CG - Control Group, SBG - Sucrose Balanced Group, AFG - Alternately Fed Group,
