# Supplementary material for: Effects of different models of sucrose intake on the oxidative status of the uterus and ovary of rats
Source: PLoS One. 2021 May 18;16(5):e0251789. doi: 10.1371/journal.pone.0251789 (PMC8130931; doi:10.1371/journal.pone.0251789)
Supplement: S6 Table — CG—Control Group, SBG—Sucrose Balanced Group, AFG—Alternately Fed Group. (DOCX) [file pone.0251789.s006.docx]

| **S6 Table.**  Effect of sucrose content diet and alternating feeding on ovarian superoxide dismutase (SOD), catalase (CAT), glutathione peroxidase (GPx) activities and malonyldialdehyde (MDA) concentrations. | | | | |
| --- | --- | --- | --- | --- |
|  |  | **CG (n=11)** | **SBG (n=11)** | **AFG (n=11)** |
| **SOD**  **(U/gHb)** | **Mean** | 10.59 | 8.99 | 8.81 |
|  | **SD** | ±0.27 | ±0.74 | ±0.23 |
|  | **Min.** | 10.00 | 7.53 | 7.02 |
|  | **Max.** | 10.99 | 9.92 | 9.05 |
|  | **Median** | 10.59 | 9.05 | 8.19 |
| **GPx**  **(U/gHb)** | **Mean** | 15.67 | 15.07 | 15.39 |
|  | **SD** | ±2.33 | ±3.20 | ±2.90 |
|  | **Min.** | 12.89 | 9.99 | 10.09 |
|  | **Max.** | 20.05 | 21.41 | 19.77 |
|  | **Median** | 15.00 | 15.78 | 15.14 |
| **CAT**  **(U/gHb)** | **Mean** | 24.46 | 21.98 | 24.78 |
|  | **SD** | ±3.67 | ±3.65 | ±4.20 |
|  | **Min.** | 18.00 | 15.70 | 16.50 |
|  | **Max.** | 29.76 | 28.99 | 33.07 |
|  | **Median** | 23.04 | 22.28 | 23.89 |
| **MDA**  **(µmol/L)** | **Mean** | 4.95 | 4.70 | 5.03 |
|  | **SD** | ±0.88 | ±1.00 | ±0.99 |
|  | **Min.** | 3.75 | 3.21 | 4.01 |
|  | **Max.** | 6.59 | 6.85 | 6.73 |
|  | **Median** | 4.99 | 4.60 | 4.68 |

CG - Control Group, SBG - Sucrose Balanced Group, AFG - Alternately Fed Group,
