# Supplementary material for: Effects of different models of sucrose intake on the oxidative status of the uterus and ovary of rats
Source: PLoS One. 2021 May 18;16(5):e0251789. doi: 10.1371/journal.pone.0251789 (PMC8130931; doi:10.1371/journal.pone.0251789)
Supplement: S7 Table — CG—Control Group, SBG—Sucrose Balanced Group, AFG—Alternately Fed Group. (DOCX) [file pone.0251789.s007.docx]

| **S7 Table.**  Effect of sucrose content diet and alternating feeding on uterine superoxide dismutase (SOD), catalase (CAT), glutathione peroxidase (GPx) activities and malonyldialdehyde (MDA) concentrations. | | | | |
| --- | --- | --- | --- | --- |
|  |  | **CK (n=11)** | **SBG (n=11)** | **AFG (n=11)** |
| **SOD**  **(U/gHb)** | **Mean** | 7.40 | 5.91 | 5.0 |
|  | **SD** | ±0.73 | ±1.00 | ±1.08 |
|  | **Min.** | 6.11 | 4.09 | 3.02 |
|  | **Max.** | 8.30 | 7.75 | 6.38 |
|  | **Median** | 7.40 | 5.89 | 5.27 |
| **GPx**  **(U/gHb)** | **Mean** | 8.67 | 8.80 | 11.05 |
|  | **SD** | ±1.04 | ±1.56 | ±1.38 |
|  | **Min.** | 7.00 | 6.80 | 8.64 |
|  | **Max.** | 10.99 | 11.90 | 12.65 |
|  | **Median** | 8.67 | 8.70 | 11.56 |
| **CAT**  **(U/gHb)** | **Mean** | 16.13 | 14.19 | 18.83 |
|  | **SD** | ±1.26 | ±1.18 | ±2.00 |
|  | **Min.** | 14.01 | 12.30 | 15.91 |
|  | **Max.** | 18.28 | 16.96 | 21.63 |
|  | **Median** | 16.36 | 14.25 | 18.83 |
| **MDA**  **(µmol/L)** | **Mean** | 2.94 | 2.40 | 3.90 |
|  | **SD** | ±0.60 | ±0.70 | ±0.90 |
|  | **Min.** | 1.91 | 1.03 | 2.57 |
|  | **Max.** | 3.93 | 3.13 | 5.12 |
|  | **Median** | 2.88 | 2.68 | 3.90 |

CG - Control Group, SBG - Sucrose Balanced Group, AFG - Alternately Fed Group,
